# Gross Cystic Disease Fluid Protein-15(GCDFP-15)/Prolactin-Inducible Protein (PIP) as Functional Salivary Biomarker for Primary Sjögren’s Syndrome

**DOI:** 10.4172/2157-7412.1000140

**Published:** 2013-06-15

**Authors:** A Gallo, D Martini, F Sernissi, C Giacomelli, P Pepe, C Rossi, PP Riveros, M Mosca, I Alevizos, C Baldini

**Affiliations:** 1 Sjögren’s Syndrome Clinic, Molecular Physiology and Therapeutics Branch, National Institute of Dental and Craniofacial Research, National Institutes of Health, USA; 2 Rheumatology Unit, University of Pisa, Italy via Roma 67, 56126, Pisa, Italy

**Keywords:** Sjören’s syndrome, GCDFP-15/PIP, Autoimmune disease

## Abstract

**Background:**

Gross cystic disease fluid protein-15(GCDFP-15)/prolactin-inducible protein (PIP) is a secretory acinar glycoprotein of 14 KDa which we have recently described as significantly lower in salivary samples of patients with primary Sjögren’s syndrome (pSS) in comparison to healthy volunteers by proteomic analysis.

**Aims of the study:**

(1) to validate our previous data on the decrease of GCDFP-15/PIP protein in a larger number of subjects with pSS (2) to integrate the proteomic results with complementary immunoassays in order better clarify the pathophysiological relevance of GCDFP-15/PIP in pSS exocrinopathy (3) to assess both the glandular expression of the GCDFP-15/PIP and the levels of glandular GCDFP-15/PIP mRNA in the patients’ minor salivary gland (MSG) biopsies in order to verify whether the observed reduction of GCDFP-15/PIP in saliva may be related to a decrease in the protein production.

**Patients and methods:**

A total of 123 salivary samples from patients affected by pSS, no-SS sicca syndrome and sex- age-matched healthy volunteers were analyzed by different proteomic techniques (SELDI-TOF-MS, 2DE, MALDI-TOF-MS). The expression of GCDFP-15/PIP was then validated by western blot analysis. Real Time PCR and immunohistochemistry for GCDFP-15/PIP in the minor salivary glands (MSG) biopsies were then carried out.

**Results:**

By using complementary proteomic analysis we found that a putative peak of 16547 m/z was among the best independent biomarkers for pSS able to discriminate between patients and healthy controls with a sensitivity of 96 % and a specificity of 70%, with a global cross validated error of 29%. We identified the peak as the GCDFP-15/PIP protein and verified that the intensity of GCDFP-15/PIP was significantly lower in pSS patients when compared to both no-SS sicca subjects and healthy controls (p<0.0001). GCDFP-15/PIP expression also correlated with both the salivary flow rate (r=0.312, p=0.023) and MSG biopsies focus score (r=−0.377, p=0.04). Finally, immunohistochemistry confirmed that GCDFP-15/PIP staining was faint in mucus acini and Real Time PCR showed that GCDFP-15/PIP mRNA was significantly lower in pSS patients when compared to both no-SS sicca subjects and healthy controls (p=0.023) thus supporting the hypothesis that the observed reduction of GCDFP-15/PIP in pSS saliva may be related to a decrease in the protein production.

**Conclusion:**

In this study by different complementary-omic techniques we confirmed the potential role of GCDFP-15/PIP as a novel biomarker for pSS. This finding might also be functionally important as GCDFP-15/PIP has previously been shown to bind to Aquaporin 5 (AQP5), a salivary gland water channel, critical to saliva formation that is known to be downregulated in pSS. It is likely that exploring the GCDFP-15/PIP/AQP5 axis will help better understand the mechanism of salivary gland dysfunction in pSS.

## Introduction

Primary Sjören’s syndrome (pSS) is a systemic autoimmune disease characterized by salivary and lachrymal glands dysfunction and inflammation, resulting in severe dry eye and dry mouth. During the last few years, mainly due to the advances of emerging high throughput genomic and proteomic technologies, several efforts have been made in searching for novel diagnostic and therapeutic biomarkers for pSS ultimately paving the way to the development of new concepts for therapies [1,2]. At present, the most consistent “–omics” data are those related to the identification of proteomic biomarkers for pSS in human saliva [3–5]. Saliva has been recognized as an attractive biological fluid for pSS since it may closely reflect the underlying glandular autoimmune exocrinopathy and can be repeatedly collected in a non-invasive manner. A number of inflammatory and salivary epithelial proteins have been seen as potential biomarkers for the disease [6–11]. Among those putative proteomic salivary biomarkers for pSS, we have developed a specific interest for gross cystic disease fluid protein-15/prolactin-inducible protein (GCDFP-15/PIP), a secretory acinar protein of 14KDa that has been identified as significantly under-expressed in salivary samples of pSS patients in comparison to healthy volunteers in previous studies carried out by our and other groups [6–9]. Although the function of GCDFP-15/PIP remains unknown, it has been reported that GCDFP-15/PIP binds to many proteins, including fibrinogen, actin, keratin, myosin, and also the CD4 molecule of T cells, thus inhibiting T-lymphocyte apoptosis [12]. In addition, recent observations in mouse models for pSS have shown that GCDFP-15/PIP may also bind to the C-terminal portion of aquaporin-5 (AQP5) leading to its physiological translocation from cytoplasm to the apical membrane of lachrymal glands [13]. Thus, an aberrant expression of GCDFP-15/PIP might negatively affect the trafficking of AQP5 from the cytoplasm to the membrane of the acinar cells subsequently interfering with the glandular secretion processes in salivary and lachrymal glands. The relevance of exploring the GCDFP-15/PIP/AQP5 axis in pSS hyposalivation might be foresee considering that for the past 15 years, our laboratory has studied the value of developing a gene transfer approach, using the Aquaporin family of genes to restore salivary flow in patients with radiation-damaged salivary glands [14–16].

The aim of this study was therefore first to validate our previous data on the decrease of GCDFP-15/PIP in a larger number of subjects with pSS and second, to integrate the proteomic results with complementary immunoassays in order to better clarify the pathophysiological relevance of GCDFP-15/PIP in pSS exocrinopathy. More specifically, we correlated salivary GCDFP-15/PIP levels with patients’ salivary flow and minor salivary gland lymphocytic infiltration, in order to explore if GCDFP-15/PIP reduction reflects the functional impairment of the salivary secretion process. We also assessed both the glandular expression of the GCDFP-15/PIP and the levels of glandular GCDFP-15/PIP mRNA in minor salivary gland (MSG) biopsies in order to verify whether the observed reduction of GCDFP-15/PIP in saliva may be related to a decrease in the protein production. Ultimately, exploring the GCDFP-15/PIP/AQP5 axis may open novel scenarios for our understanding of salivary gland hypofunction in pSS.

## Materials and Methods

### Patients and experimental design

**Figure 1:**
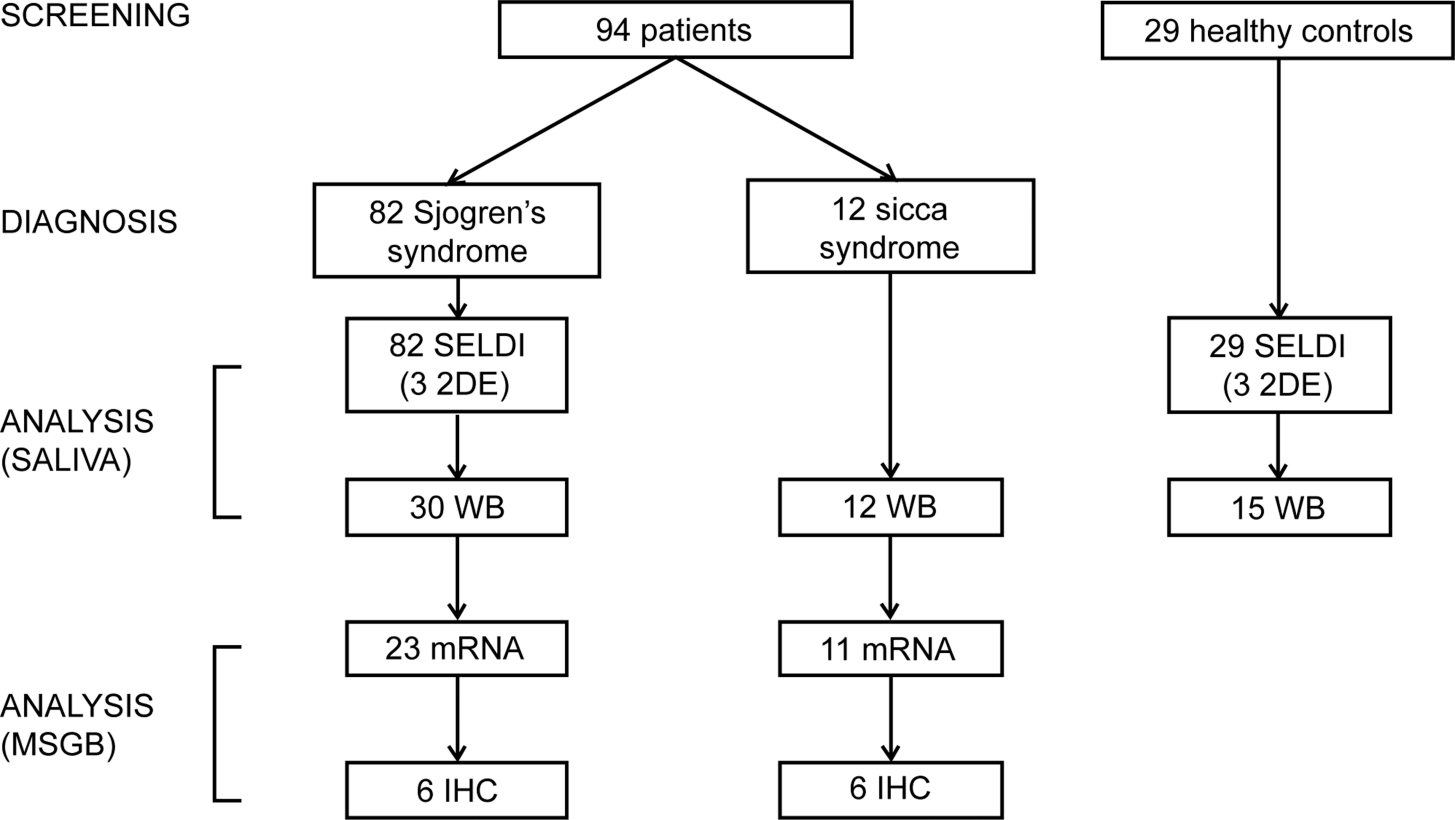
Flow chart of the patients’ enrolled in the study (2DE, 2D electrophoresis; WB, western blot; MSGB, minor salivary gland biopsies; IHC, immunohistochemistry).

This study was approved by the ethics committee of our academic hospital. A total of 123 salivary samples from patients affected by pSS, no-SS sicca syndrome and sex- and age-matched healthy volunteers were collected consecutively from May 2012 to February 2013 at the Rheumatology Unit of the University of Pisa. The diagnosis of pSS was made according to the International Classification Criteria for the disease (AECG) [17] while the condition of no-SS sicca syndrome was defined as the presence of xerostomia in patients who did not meet the diagnostic AECG criteria for SS (non-SS sicca, CTL). To be included in the study all the subjects had to be at least 18 years old and able to provide a signed informed consent. According to the AECG, exclusion criteria for pSS patients included: past head and neck radiation treatment, hepatitis C infection, acquired immunodeficiency disease (AIDS), pre-existing lymphoma, sarcoidosis, Graft versus host disease, and use of anticholinergic drugs. Detailed medical charts were available for all patients. Variables analyzed in the study included: sex, age, time since diagnosis, subjective dry eyes and mouth, and parotid gland enlargement. Minor salivary gland (MSG) biopsy results were classified according to the focus scoring system. Patients’ treatments at the inclusion in the study were also recorded. At the time of the study entry, every patient had an evaluation of salivary flow rate, ophthalmological examination and autoantibody testing of the serum (i.e antinuclear antibodies, extractable nuclear antigen (ENA) antibodies, rheumatoid factor, anticentromere auto-antibodies (ACA), anti Scl-70, anti-cyclic citrullinated peptide (anti-CCP)). Salivary samples were collected under standard conditions and analyzed using different proteomic techniques (SELDI-TOF-MS, 2DE, MALDI-TOF-MS). The data generated from the proteomic analysis were then validated by western blot analysis. This second validation phase also included the analysis by Real Time PCR and immunohistochemistry of the expression of GCDFP-15/PIP in the MSG biopsies obtained for diagnosis from subjects who had been newly diagnosed with pSS- or no-SS sicca syndrome (Figure 1).

### Samples collection and processing

#### Saliva samples

Unstimulated whole salivary samples were collected between 9 and 11 a.m. from patients who had refrained from eating or drinking for 2 h following previously described procedures [7]. Immediately after the collection and salivary flow rates determination in order to minimize the degradation of the proteins, the samples were processed immediately and kept on ice during the entire process. Samples were immediately centrifuged at 13.000 × g for 20 minutes at 4°C to remove debris and cells, and protein amounts of resulting supernatants were determined using Protein Assay dye reagent (Bio-Rad; Richmond, CA). Aliquots of the samples were stored at −80°C until analysis.

#### Minor salivary gland biopsy samples

Minor salivary glands (MSGs) were obtained as part of routine diagnostic procedures when pSS was suspected. MSG biopsies were performed after local anesthetic infiltration. In all the cases, the MSG specimens from each subject were divided and some were fixed in neutral buffered formalin for the focus score assessment, and the rest were snap frozen and stored at −80°C. All of the MSG formalin-fixed specimens were processed (paraffin embedding, sectioning and hematoxylin and eosin staining) and evaluated by the same pathologist. If a diagnosis of focal lymphocytic sialoadenitis was made, the focus score was then determined according to the scoring guidelines [18]. The frozen sample was employed for the Real time PCR analysis.

### SELDI-TOF analysis

The ProteinChip System, Series 4000 Personal Edition (Ciphergen Biosystems, Inc) was used to perform SELDI-TOF MS. After preliminary experiments with different protein chip arrays (including Q10, IMAC30 and H50, Bio-Rad), the cation exchange array CM10 (Bio-Rad) - a weak cation exchange chip - was selected to give the best results and prepared according to the manufacturer’s instructions. Briefly, 20 µg of proteins, previously treated with 2/3 (v/v) denaturing buffer (CHAPS 2%, Urea 9M) were directly applied to each Protein Chip array spot. The experiment was conducted as previously described [19,20]. Chips were read on a Ciphergen Express Data Manager – Personal Edition (version 3.5).

### 2DE analysis and mass spectrometry analysis

Two-dimensional electrophoresis (2DE) was performed using the Immobiline-polyacrylamide system with pH 3–10L, 17 cm IPG strips (Protean IEF Cell, Bio-Rad). The second dimension (SDS-PAGE) was performed using 15% polyacrylamide gels, maintained at 12°C. The analytical gels were stained with colloidal Coomassie Silver Blue [21] and scanned using the GS800 Densitomer (Bio-Rad). Subsequently, the gels were analyzed with the PDQuest advanced software. Spots of interest were cut out from the master gel and MALDI-TOF mass spectrometry technique was used to identify the spots of interest, cut out from the master gel as previously described [6,7]. In-gel digestion and mass spectrometric (MS) analyses were performed at the Mass Spectrometry Facility (Biotechnology Center, University of Wisconsin-Madison) as outlined on the Center website, as previously described (http://www.biotech.wisc.edu/ServicesResearch/MassSpec/ingel.htm).

### Western blot analysis

Western Blot (WB) analysis was used validate the 2DE results. We employed the Stain-Free technology which has recently demonstrated to be a more reliable, more robust, and more sensitive normalization tool for WB experiments when compared to traditional housekeeping protein (HKP) normalization [22]. More specifically, one-dimensional SDS-electrophoresis was first performed. Briefly, aliquots of all saliva samples (30 µg of total protein) were treated with Laemmli buffer and heated at 100°C for 5 minutes, loaded on 4–20% Mini-PROTEAN TGX Stain-Free (#456–8093, Bio-Rad), and run for 30 minutes at 200 V, according to manufacturer’s instructions. After electrophoresis, stain-free gels underwent UV activation, as previously described [22] in order to assess protein transfer after blotting (total protein volume acquired from each lane by ChemiDoc, Bio-RAD). WB was subsequently performed, as previously described [23], using an N-terminal monoclonal anti-human GCDFP-15/PIP antibody (Abcam; Cambridge, UK) as primary antibody. The chemiluminescence expressed in terms of volume of specific immunoreactive bands was determined (ChemiDoc, Bio-Rad), and normalized to the total protein volume previously acquired. Each value was then normalized on an internal control sample (the same for every gel) treated in the same experimental conditions, in order to compare samples which run in different gels.

### RNA extraction and pPCR

Total RNA was extracted from frozen minor salivary gland biopsy: tissues were disrupted and homogenized through high-speed shaking with beads (Qiagen) using the TissueLyser (Qiagen) and RNA was purified by miRCURY RNA Isolation kit (EXIQON) according to manufacturer’s instructions. The RNA reverse-transcription was carried out using 500 ng of total RNA using High Capacity cDNA Reverse Transcription Kit (Applied Biosystems). Taqman Probe for the GCDFP-15/PIP mRNA and GAPDH mRNA were used (part no. Hs00160082_m1 and part no. Hs02758991_g1, respectively). Real Time PCR was performed in triplicate on an Eco real time instrument (Illumina Inc) following the standard protocol. Transcripts were evaluated by TaqMan Gene Expression Assays (Applied Biosystems): 20ul PCR was run with cycling conditions of 10 minutes at 95°C, followed by 40 cycles of denaturing for 15 seconds at 95°C, and annealing and extending for 60 seconds at 60°C. Amplifications were normalized by GAPDH (Hs02758991_g1) and quantitation of gene expression was performed using the ΔΔCT calculation, where CT is the threshold cycle. The amount of the target gene normalized and relative to the calibrator (control sample) is given as 2- 2- ΔΔCT.

### Immunohistochemistry analysis

Rehydrated 5-µm paraffin sections of formalin-fixed salivary gland biopsies, were pretreated using high temperature antigen retrieval in Citrate buffer 0.01M, pH 6 for 15 min at 80% power, and brought it to RT for 15 min. An incubation of 20 min in 100 mM glycine at RT was used to block the free aldehydes. To block nonspecific binding 10% FBS, 0.4% saponin, 0.02% NaN3 in PBS in PBS buffer pH 7.4 was used. The sections were incubated with the primary antibody anti- GCDFP15 (ab62363, Abcam) diluted in blocking buffer 1/200 overnight at 4°C. For the secondary fluorescence-labeled antibodies, 1:300 dilution of AlexaFluo 488-conjugated (red) or Alexa 594–conjugated (green) antibodies were used (Jackson Immuno Research; West Gorve, PA). The bands were visualized with Histostain^®^-Plus 3^rd^ Gen IHC Detection Kit (Invitrogen, CARLSBAD, CA) according to the manufacturer. For nuclear counterstain Mayer’s Hematoxylin was used. Finally the slides were coversliped using CitraMount medium (Electron Microscopy Hatfield, PA).

### Statistical analysis

#### SELDI_TOF_MS data analysis and CART algorithm

On the basis of their distributional assumptions, SELDI-TOF-MS data were analyzed by using a non-parametric approach which was carried out by Mann-Whitney test for univariate analysis coupled with a classification and regression tree (CART) algorithm in the multivariate setting. Only significant variables (p<.05) at the univariate analysis were included in the CART algorithm.

The construction of CART included two steps, namely the tree construction step and the tree pruning step. For the tree construction process, the best peak was searched with defined cut-off level so that the dataset was split into two daughter nodes. The splitting decision was based on the peak intensity of a sample. Samples went to the left daughter node if their peak intensities were equal to or less than the cut-off intensity value; otherwise, the samples would go to the right daughter node. instead of Samples went to the left daughter node if their peak intensities were equal to or less than the cut-off intensity value; otherwise, the samples would go to the right daughter node. The software continued to repeat this splitting process on each daughter node in this manner until no further gain in the classification was achieved and terminal nodes were produced. Classification of the terminal nodes was decided by a group of samples, which represented the majority of samples in that group. In the second step of CART, the classification tree was cut down to a desired size that yielded the least classification error. The best among all pruned trees were selected on the basis of the minimization of the leave one out cross validation error. Statistical analysis was performed by using” R: A language and environment for statistical computing. R Foundation for Statistical Computing, Vienna, Austria. ISBN 3-900051-07-0, URL http://www.R-project.org/.”

### Statistical analysis of WB and qPCR results

Continuous data were expressed as median and interquartile range. Statistical analyses of the results were carried out using the χ2 test and the Mann–Whitney test. Spearman’s rank correlation coefficient was employed in order to correlate subjects’ clinical parameters with the normalized values of band intensity from Western blot and the log of RQ values from RT-PCR. In addition a Semi-Quantification of the normalized values of GCDFP-15/PIP band intensity from Western blot (normal or low) was also obtained using the minimum value of GCDFP-15/PIP expression level of normal controls as a cut-off point. The p-values <0.05 were considered significant. Statistical analysis software SPSS version 16.0 (SPSS Inc., Chicago, IL, USA) was used for statistical analysis.

## Results

### Patients

Unstimulated whole saliva samples were collected from 89 consecutive unselected female patients with pSS (age at inclusion, mean (SD) = 54.6 yrs (13.8 yrs) and from 29 sex- and age- matched HC (age at inclusion, mean (SD) = 53.2 yrs (12.8 yrs)) and analyzed using complementary proteomic techniques (i.e. SELDI-TOF-MS and 2DE/MALDI-TOF-MS) (Figure 1). Unstimulated whole salivary flow rates of the groups (median and IQR) were 2 ml/15 min (1.1 to 4.125 ml/15min) and 4.15 ml/15 min (2.5 to 7.875 ml/15 min) for pSS patients and HC, respectively (p=0.001). Of the 89 pSS patients, 30/89 was newly diagnosed with pSS: these patients were included also in the second phase of the study and their MSG biopsies were collected and analyzed as well. The control group for this second phase of the study consisted of 15/29 HC and 12 no-SS sicca syndrome subjects, both recruited during the same period of the 30 pSS patients (Figure 1). All the no-SS sicca syndrome subjects underwent MSG biopsy. Subjects’ demographic and clinical features are summarized in Table 1.

### SELDI-TOF MS results

**Figure 2:**
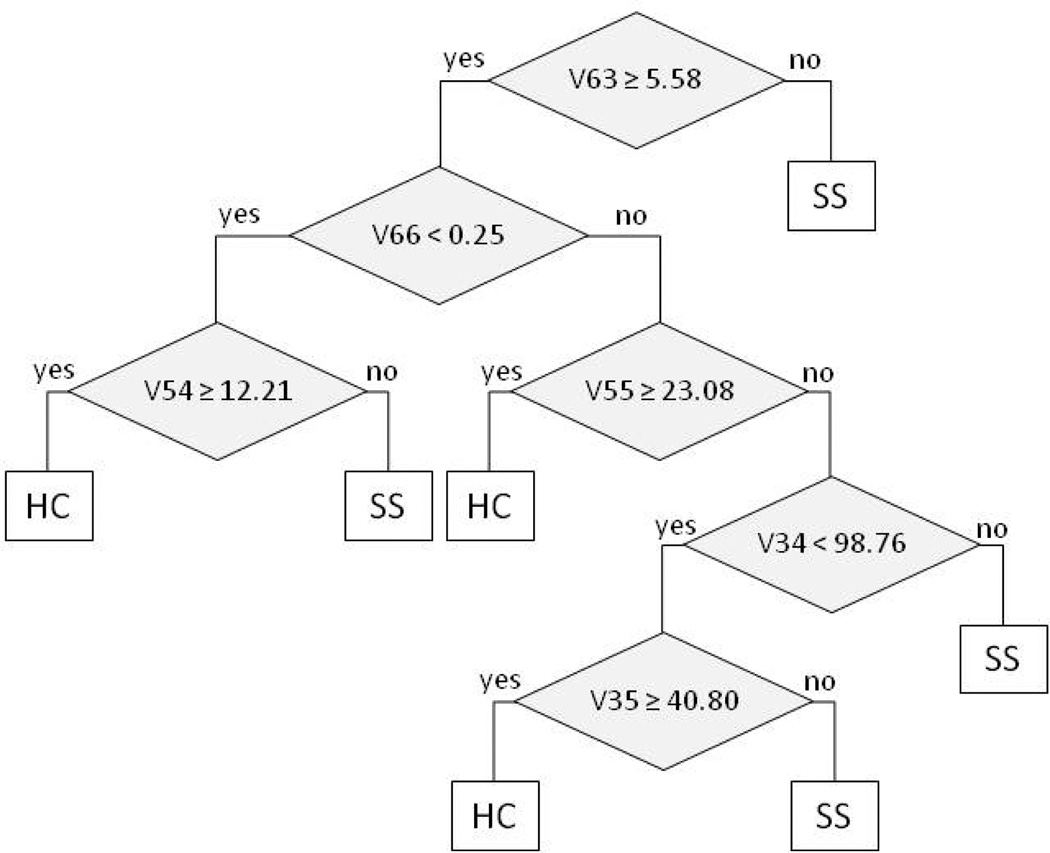
Classification and regression tree (CART) algorithm, as result of the analysis of the SELDI-TOF-MS results (SS, Sjögren’s syndrome; HC, healthy control, V63=16547 m/z, V66=24059 m/z, V54=13507 m/z, V55=13714 m/z, V34=7149 m/z, V35=7192 m/z).

A total of 75 peaks were detected. Peak values were generated for each sample. Mean peak intensities of the groups were compared by Mann Whitney test at the univariate analyses. We found that 25 peaks were significantly different in the pSS patient group with respect to non-SS (*p*<0.05). Among these 25 peaks, the selected CART tree indentified 7149 m/z (v34), 7192 m/z (v35), 13507 m/z (v54), 13714 m/z (v55), 16547 m/z (v63), 24059 m/z (v66) as best independent biomarkers able to discriminate between pSS and HC with a sensitivity of 96 % and a specificity of 70%, with a global cross validated error of 29% (Figure 2).

### Identification of GCDFP-15/PIP protein

**Figure 3:**
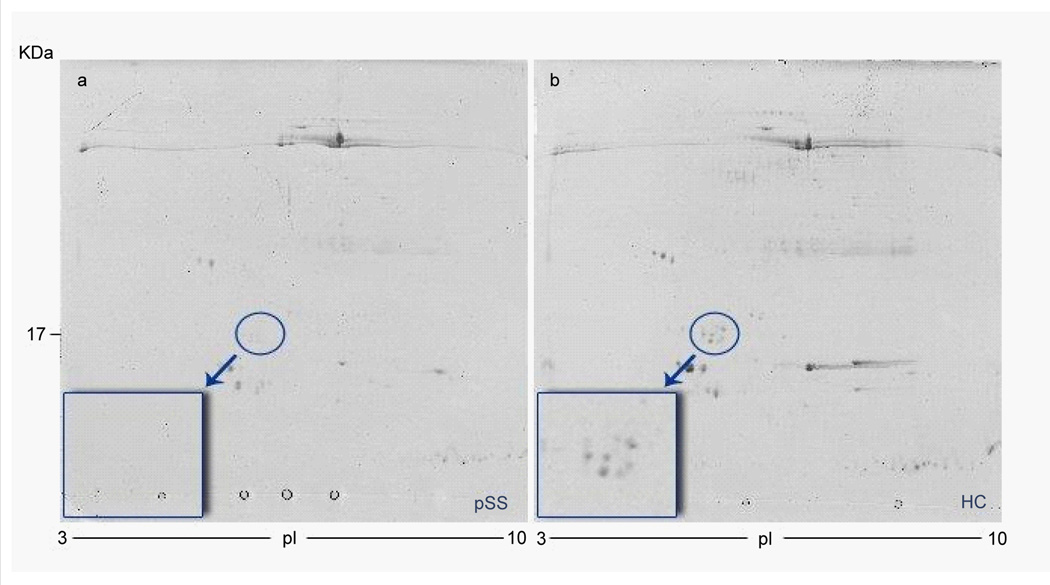
Representative 2DE - gel map from a) primary Sjögren’s syndrome patient b) healthy control.

We specifically focused on the 16547 m/z peak, which splitting the dataset in two daughter nodes yielded the least classification error. This peak was significantly reduced in pSS vs HC (p<0.0001). We thought that this 16547 m/z peak might be potentially related to GCDFP-15/PIP protein (calculated mass 16572 Da) which has been previously described as significantly decreased in pSS saliva. In order to verify our hypothesis we compared SELDI-TOF MS and 2DE results for the same salivary samples as in previous studies [20,24]. 2DE analysis was performed with 3 salivary samples from each of the two groups (pSS and HC) and the identification of the putative spots related to the GCDFP-15/PIP protein was performed by MALDI-TOF-MS (mascot: http://www.matrixscience.com/: accession no. P12273; Nominal mass (Mr): 16562; calculated pI value: 8.26; sequence coverage: 46%; score 229). We obtained similar profiles in SELDI analyses and in the 2DE for each of the samples tested (Figure 3).

### Western Blot analysis

In order to further validate our work hypothesis and to better investigate the diagnostic role of GCDFP-15/PIP as putative diagnostic biomarker, we assessed the salivary expression levels of GCDFP-15/PIP by WB in patients newly diagnosed with pSS, HC and in patients of the sicca group no-SS sicca who had been recruited during the same time period of pSS patients.

**Figure 4:**
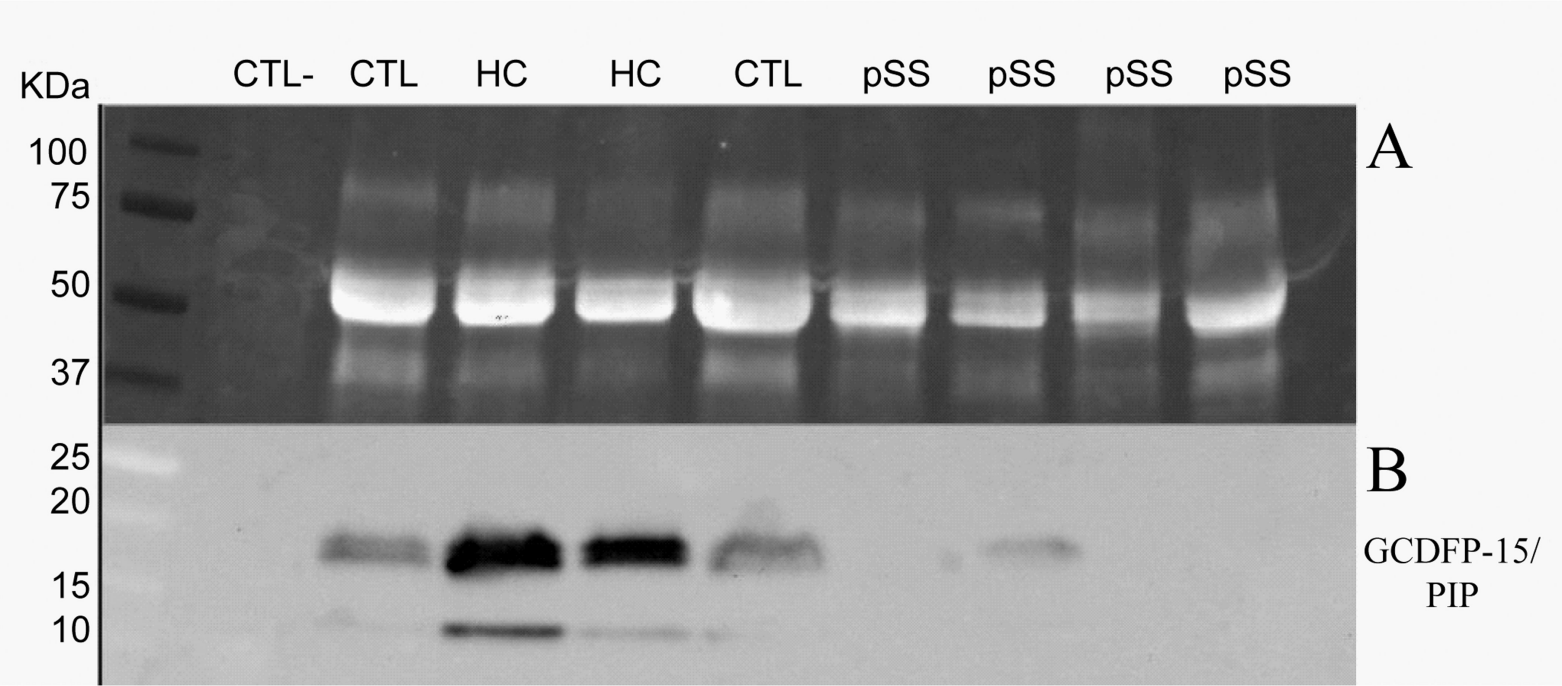
Median and interquartile range of GCDFP-15/PIP optical density of patients and healthy controls (p<0.0001 by ANOVA analysis). GCDFP-15/PIP is differently expressed between primary Sjögren’s syndrome patients (pSS) and non-SS sicca syndrome patients (CTL), with p=0.01 (*), and between pSS and healthy control subjects (HC), with p<0.0001 (***).

**Figure 5:**
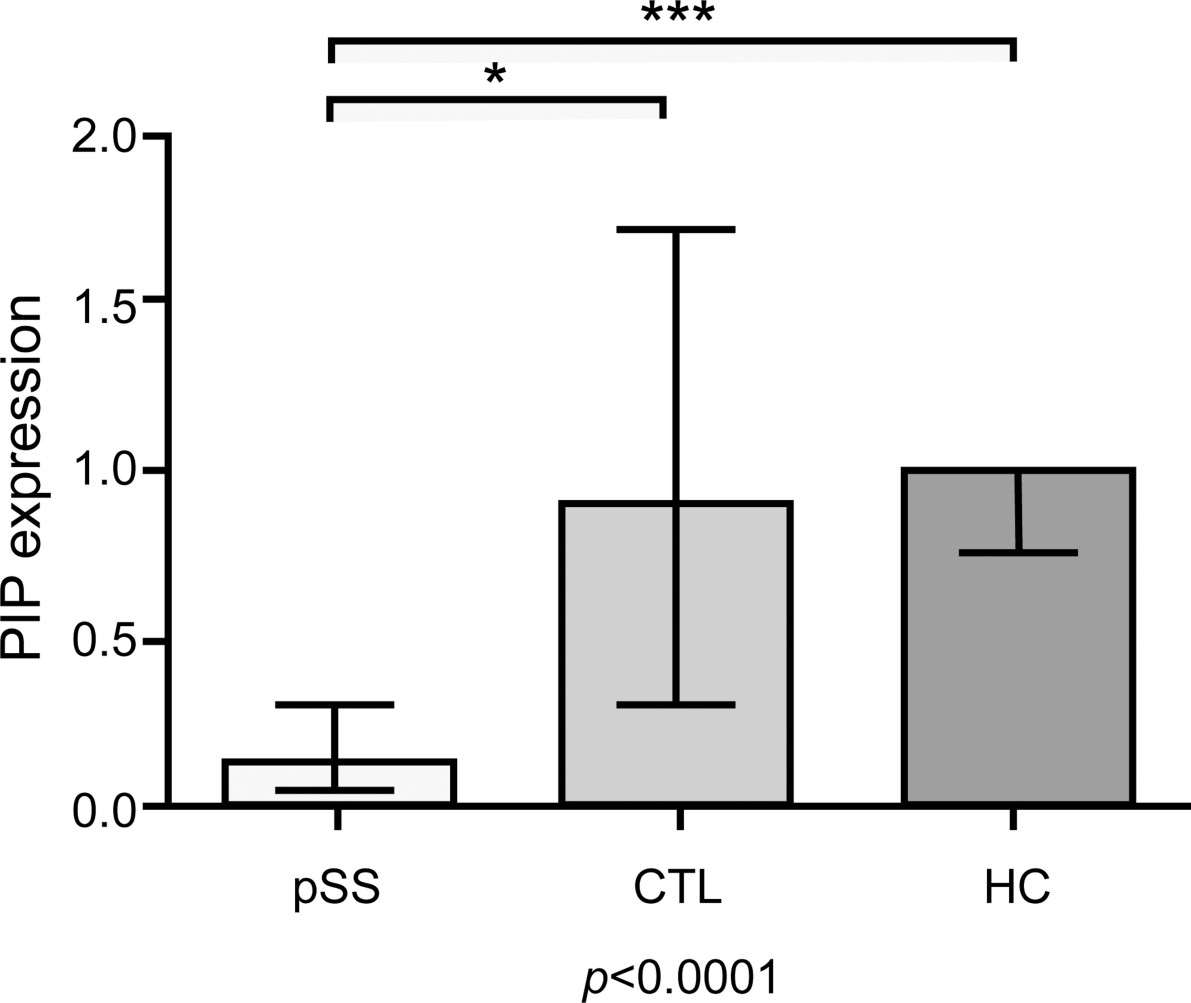
Representative Western blot comparative analysis between Sjögren’s syndrome patients (pSS), non-SS sicca syndrome patients (CTL), and healthy control subjects (HC). a) acquisition of total protein content run during 1D SDS-electrophoresis, after the stain-free gel was UV-activated; b) acquisition of immunoreactive GCDFP-15/PIP band on a nitrocellulose film.

Comparison between the pSS group and control groups disclosed that the intensity of GCDFP-15/PIP was significantly lower in pSS patients when compared to both no-SS sicca subjects and HC using Anova test and Bonferroni correction post-hoc test (p<0.0001). Figure 4 shows a representative WB of GCDFP-15/PIP on salivary protein extract, while the Figure 5 represents the Boxplot of GCDFP-15/PIP expression measured by WB in the three groups (median and interquartile range). We also found that GCDFP-15/PIP expression correlated with both the salivary flow rate (r=0.312, p=0.023) and MSG biopsy focus score (r=−0.377, p=0.04).

**Figure 6:**
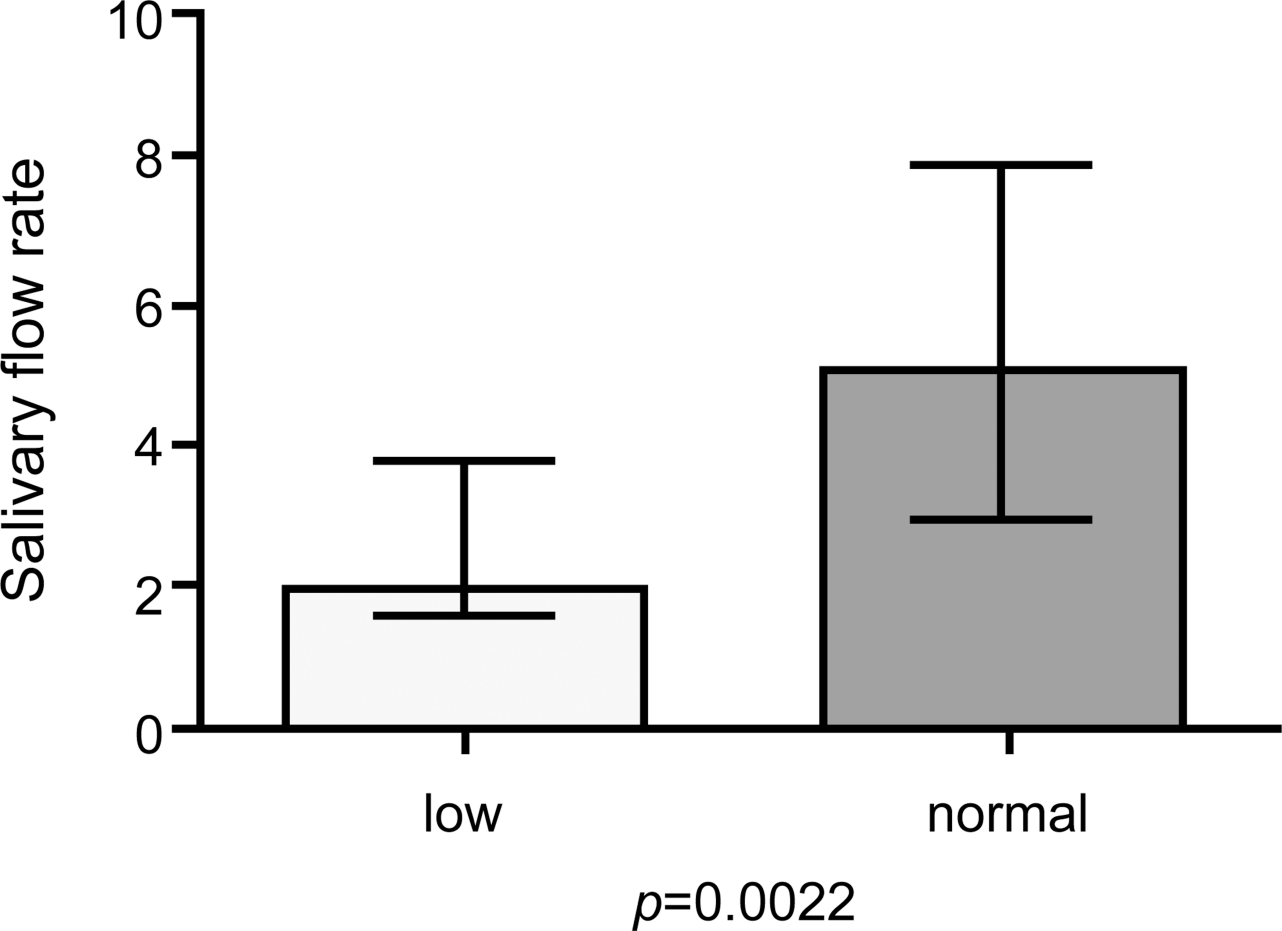
Difference of salivary flow rate between patients with low versus normal GCDFP-15/PIP expression, as result of a qualitative western blot analysis, obtained by using the minimum value of GCDFP-15/PIP expression level of healthy controls as a cut-off point to discriminate between a low and a normal expression (Mann Whitney test, p=0.0022).

Using the minimum value of GCDFP-15/PIP expression level of normal controls as a cut-off point we obtained a semiquantification of the GCDFP-15/PIP protein levels measured by Western Blot. This allowed us not only to confirm the different expression of GCDFP-15/ PIP proteins between the groups (p<0.0001) but also to correlate GCDFP-15/PIP levels with some of the subjects clinical features. More specifically we found that the decrease of GCDFP-15/PIP salivary expression was directly correlated with both the reduction of the salivary flow rate (p=0.002) and with the focus score of the MSGBs (p=0.023). Figure 6 shows the Boxplot of the salivary flow rate when patients were separated into two groups according to their WB expression level of GCDFP-15/PIP (normal or low), using the minimum value of expression level of normal controls as a cut-off point.

### mRNA GCDFP-15/PIP in MSGs

**Figure 7:**
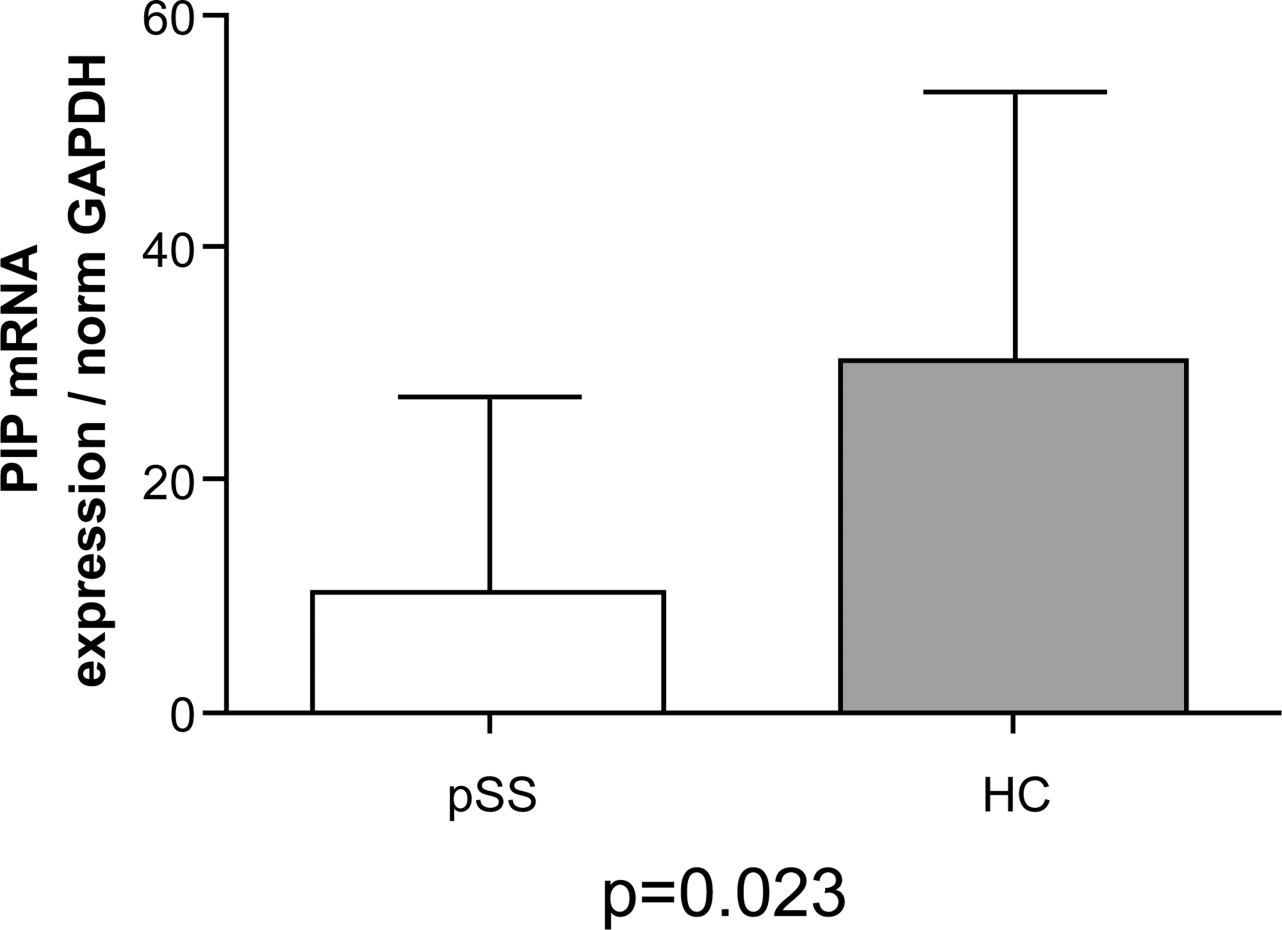
Difference of mRNA expression (GCDFP-15/PIP mRNA expression normalized on GAPDH) between patients with primary Sjögren’s syndrome (pSS) and no-SS sicca syndrome subjects (CTL) (Mann Whitney test, p=0.023).

To investigate whether the mRNA level of GCDFP-15/PIP were lower in PSS than in the sicca group according to the protein level trend, real-time PCR was conducted on the RNA extracted from salivary gland biopsies. 23 pSS and 11 control salivary gland biopsies were lysed and total RNA exctracted and retrotrascribed. The mRNA levels of GCDFP-15/PIP were normalized to GAPDH mRNA level. Comparison of the GCDFP-15/PIP mRNA level between the pSS group and the control groupshowed that GCDFP-15/PIP mRNA was significantly lower in pSS patients when compared to no-SS sicca subjects using Mann Whitney post hoc test (p=0.023). Figure 7 represents the Boxplot of GCDFP-15/PIP expression measured by pPCR in the two groups (median and interquartile range). We also found that GCDFP-15/PIP mRNA expression correlated with both the salivary flow rate (r=0.676, p=0.002) and MSGBs’ focus score (r=−0.379, p=0.03).

### Immunohistochemistry

**Figure 8:**
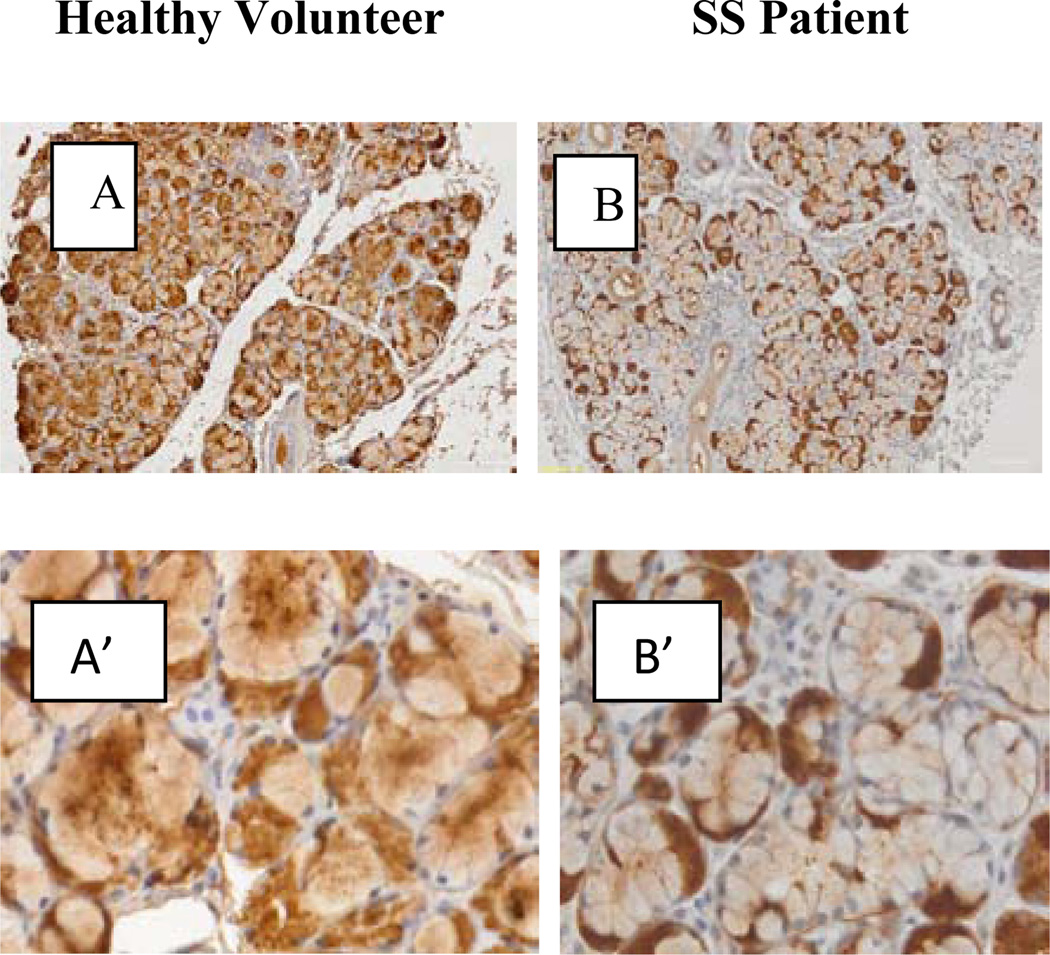
Immunohistochemical studies of GCDFP-15/PIP in sections derived from SS biopsies and healthy volunteer. In healthy volunteer the staining is more concentrated towards the apical pole of mucuse cells (acinous marked with m in figure 8A’) and ductal cell are negative, is also possible observe some immunorective material in the lumen of mayor ducts (asterisk in figure 8A). In SS patients the immunoreactivity for GCDFP-15/PIP is faint in mucus acini (see m in figure 8B’), no major changes were observed in serous acinar cells but slightly increase in the intensity of the immunoreactivity in this cells.

Immunohistochemical studies of GCDFP-15/PIP in sections derived from pSS biopsies andno-SS sicca controls, showed that the staining of GCDFP-15/PIP is located intracellular in mucus and serous acinar cells (Figure 8). In controls the staining is more concentrated towards the apical pole of mucous cells (marked with m in Figure 8A’) and ductal cell are negative. In some cases it is also possible to observe some immunoreactive material in the lumen of major ducts (asterisk in Figure 8A). Compared to the controls, in SS patients the immunoreactivity for GCDFP-15/PIP is faint in mucous acini (m in Figure 8B’) and no major differences were observed in serous acinar cells (Figure 8B).

## Discussion

This study, integrating emerging and complementary techniques, allowed us to investigate, simultaneously in saliva and in MSG biopsies, the potential role of GCDFP-15/PIP as a novel biomarker for pSS. We first demonstrated by SELDI-TOF-MS that a 16547 m/z peak was the best independent biomarker for pSS yielding the least global cross validated error with a good sensitivity and specificity. SELDI-TOF-MS technique does not allow a direct identification of any putative peak/biomarker but only indirect identifications. Thus, we decided to verify the potential correspondence between the 16547 m/z peak and GCDFP-15/PIP protein since, among all the candidate proteins, GCDFP-15/PIP was the closest in terms of molecular weight to the detected peak and it has been previously described as significantly reduced in pSS. The parallel analysis of the same salivary samples by SELDI-TOF-MS and 2DE/MALDI-TOF-MS strongly supported our work hypothesis and encouraged the subsequent validation of the results obtained in this first part of the study. Therefore, we validated by WB analysis the expression of GCDFP-15/PIP in the subgroup of patients newly diagnosed with pSS and we confirmed that GCDFP-15/PIP was significantly reduced in pSS patients compared to healthy controls and with those subjects originally suspected to have pSS who, at the end of the diagnostic algorithm, were diagnosed as no-SS sicca syndrome.

In the subgroup of patients newly diagnosed with pSS we were also able to assess both the glandular expression of the GCDFP-15/PIP and the levels of glandular GCDFP-15/PIP mRNA in MSG biopsies. We verified that in pSS GCDFP-15/PIP staining was faint in mucous acini and that the observed reduction of GCDFP-15/PIP in saliva was related to a decrease in the protein production rather than to an altered release of the protein. From this point of view, it is noteworthy that the mRNA levels of GCDFP-15/PIP and the salivary levels of the protein correlated with both the MSG biopsy focus score and the patients’ salivary flow rate. These results allowed us to speculate that the observed GCDFP-15/PIP salivary reduction may closely mirror pSS lymphocytic-mediated acinar damage and consider the hypothesis that the impairment of the AQP5–GCDFP-15/PIP axis might have a role in pSS glandular dysfunction [25–28]. The analysis of AQP5 distribution in MSG biopsies from pSS patients and controls was beyond the aims of this study. However, our results provide evidence which suggests that the production of GCDFP-15/PIP is significantly reduced in pSS making this protein a putative candidate biomarker for pSS diagnosis and shedding new light on the possible pathophysiological role of the protein in pSS exocrinopathy.

Taken together, these findings encourage further research in large clinical studies aimed at defining individual cut-off values for GCDFP-15/PIP protein as a novel salivary diagnostic biomarker for pSS. It is likely that once GCDFP-15/PIP abnormal levels will be defined, the protein could be routinely measured by less expensive procedures and might provide physicians with feasible information about the disease state improving patients’ overall diagnostic assessment.

In summary, this study represents a step forward in the direction of the search for novel reliable, early and non-invasive diagnostic biomarkers in autoimmune diseases through the extensive integration of genomic and proteomic studies. The expression of GCDFP-15/PIP was apparently able to reflect both the anatomical damage and the functional impairment of the salivary glands in pSS. Thus, further studies are warranted in order to better investigate the implication of the axis GCDFP-15/PIP /AQP5 on the pathogenesis of pSS sicca syndrome. Hypothetically, GCDFP-15/PIP might represent not only novel putative early non-invasive diagnostic biomarkers for the disease but also a novel specific target for gene therapy.

## Figures and Tables

**Table 1:** Patients’ demographic and clinical features (SD, standard deviation; F, female; n.s., not significant; n.a., not applicable, ANA, anti-nuclear antibodies).

First phase of the study				Second phase of the study			
	pSS (n=82)	HC (n=29)	p-value	pSS (n=30)	HC (n=15)	Non-SS (n=12)	p-value
Sex	82F	29F		30F	15F	12F	
Age at the inclusion (yrs), mean (SD)	54.6 (13.8)	53.2 (12.8)	n.s.	53.9 (12.7)	52.6 (12.4)	54.0 (13.4)	n.s.
Salivary flow rate ml/15’ (median and interquartile) (mean(SD))	2(1.1 to 4.125)3.0(2.7)	4.15(2.5 to 7.875)5.3(3.7)	0.005	2.5(1.5 to 4.25)	5.5(4.2 to 10)5.1(4.2)	3(2.1 to 6.75)6.6(3.1)	0.013
Focus score (high/low)	20/50	n.a.	n.a.	7/16	n.a.	n.a.	n.a.
ANA (%)	100	n.a.	n.a.	100	n.a.	58	0.0009
Anti-Ro/SSA (%)	57.3	n.a.	n.a.	60	n.a.	n.a.	n.a.
Anti-La/SSB (%)	22	n.a.	n.a.	18	n.a.	n.a.	n.a.
Rheumatoid Factor (%)	43	n.a.	n.a	43	n.a.	n.a.	n.a.
